# Effect of Adjuvants on Responses to Skin Immunization by Microneedles Coated with Influenza Subunit Vaccine

**DOI:** 10.1371/journal.pone.0041501

**Published:** 2012-07-25

**Authors:** William C. Weldon, Vladimir G. Zarnitsyn, E. Stein Esser, Misha T. Taherbhai, Dimitrios G. Koutsonanos, Elena V. Vassilieva, Ioanna Skountzou, Mark R. Prausnitz, Richard W. Compans

**Affiliations:** 1 Department of Microbiology and Immunology and Emory Vaccine Center, Emory University School of Medicine, Atlanta, Georgia, United States of America; 2 School of Chemical and Biomolecular Engineering, Georgia Institute of Technology, Atlanta, Georgia, United States of America; Federal University of São Paulo, Brazil

## Abstract

Recent studies have demonstrated the effectiveness of vaccine delivery to the skin by vaccine-coated microneedles; however there is little information on the effects of adjuvants using this approach for vaccination. Here we investigate the use of TLR ligands as adjuvants with skin-based delivery of influenza subunit vaccine. BALB/c mice received 1 µg of monovalent H1N1 subunit vaccine alone or with 1 µg of imiquimod or poly(I:C) individually or in combination via coated microneedle patches inserted into the skin. Poly(I:C) adjuvanted subunit influenza vaccine induced similar antigen-specific immune responses compared to vaccine alone when delivered to the skin by microneedles. However, imiquimod-adjuvanted vaccine elicited higher levels of serum IgG2a antibodies and increased hemagglutination inhibition titers compared to vaccine alone, suggesting enhanced induction of functional antibodies. In addition, imiquimod-adjuvanted vaccine induced a robust IFN-γ cellular response. These responses correlated with improved protection compared to influenza subunit vaccine alone, as well as reduced viral replication and production of pro-inflammatory cytokines in the lungs. The finding that microneedle delivery of imiquimod with influenza subunit vaccine induces improved immune responses compared to vaccine alone supports the use of TLR7 ligands as adjuvants for skin-based influenza vaccines.

## Introduction

Seasonal influenza vaccination is currently recommended in the United States for all population groups including high risk populations such as elderly or immunocompromised individuals [1]. Due to antigenic variation in viral glycoproteins and limited duration of immunity, annual vaccination is required to maintain protective immunity. To reduce the burden of re-vaccination during pandemics or seasonal drift of vaccine strains, vaccine efficacy can be enhanced by using alternative routes of immunization or by the addition of adjuvants to vaccine formulations.

The use of adjuvants with licensed influenza vaccines has focused on oil-in-water emulsions such as MF59™. Vesikari et al. demonstrated the enhanced immunogenicity of MF59 adjuvanted trivalent influenza vaccine in young children [2]. In addition, use of MF59 with avian influenza viruses (H5N1) also showed enhancement of the immune response in adults including the elderly [3]. In the United States, the only approved adjuvants for use in vaccines are aluminum hydroxide, aluminum phosphate, potassium aluminum sulfate (alum) and AS04, which contains both alum and monophosphoryl lipid A [4], [5]. In Europe, the adjuvant MF59 (oil-in-water emulsion) has been approved for use in vaccines since the 1990’s [5].

Increasingly, much work has begun to focus on adjuvants which signal through pattern recognition receptors (PRRs) including Toll-like receptors (TLRs). TLR ligands such as lipopolysaccharide, bacterial flagellin, poly(I:C) and imiquimod provide stimulation to the innate immune system resulting in the upregulation of CD80/86, production of IL-12, and increased MHC II expression [6]–[9]. Upregulation of costimulatory molecules and production of cytokines by matured dendritic cells play an important role in efficient stimulation of antigen-specific naïve lymphocytes and activation of the adaptive immune response.

Skin-based vaccinations have been shown to be an effective immunization route for a variety of pathogens. Previously, intradermal immunization using seasonal influenza vaccine has demonstrated 5-fold dose-sparing effects [10]. However, this route of skin vaccination relied on the use of the Mantoux injection method, which is known to be technically difficult [11]–[13]. Recent studies have introduced more reliable devices for intradermal injection of influenza vaccine [14]. Our labs have demonstrated that the use of microneedles patches coated with influenza vaccine antigens results in the induction of protective immune responses in animal models. Furthermore, this vaccination route induces immune responses that are equal to if not better than traditional needle based routes [15]–[17].

The types of adjuvants delivered to the skin previously include poly[di(carboxylatophenoxy)phosphazene] (PCPP) [18], CpG oligonucleotides (TLR9 ligands) [19], trimethyl chitosan [20], alum [21], QS-21 [22] and bacterial endotoxins, such as cholera toxin or heat labile toxin [23], [24]. However, little work has been reported to evaluate the effectiveness of TLR3 or 7 ligands when delivered into the skin via microneedle patches.

In the current study, we have compared the skin delivery of adjuvanted influenza subunit vaccine with coated microneedles using imiquimod or poly(I:C), both mimics of viral RNA intermediates. We have compared the immune responses, HAI and microneutralization titers as well as frequencies of IFN-γ+ effector helper T cells. The effects of the adjuvanted vaccine on protection against lethal challenge with the homologous virus were also compared to those of vaccine alone. This report highlights the first work describing the delivery of TLR3 and TLR7 ligand adjuvants by coated microneedles to the skin with an influenza subunit vaccine.

## Results

### Skin Delivery of Adjuvanted Influenza Subunit Vaccine Increases Humoral Immune Response

**Figure 1:**
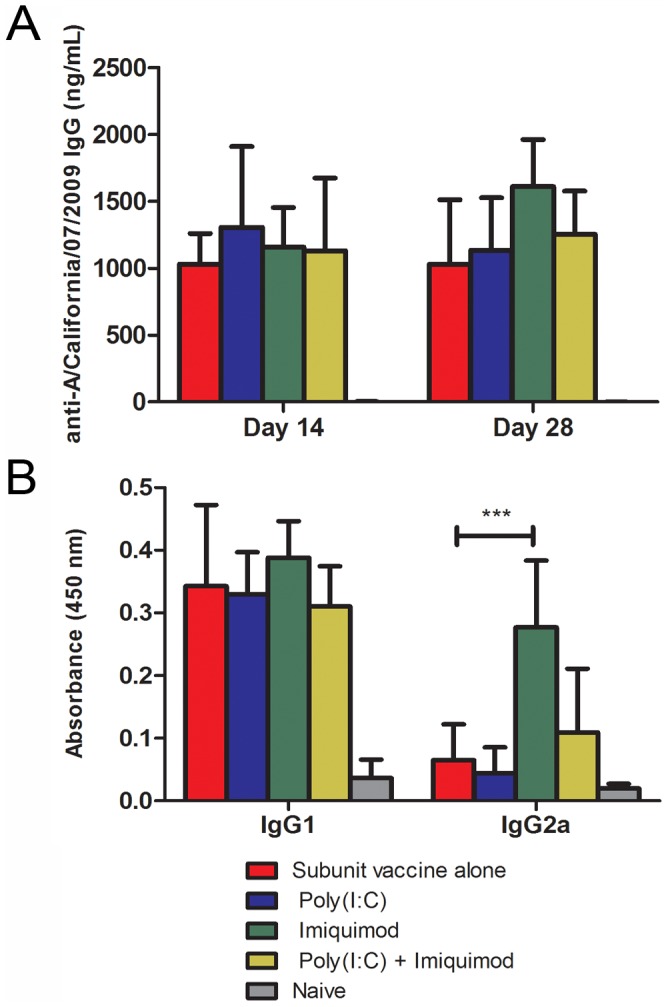
Microneedle immunization with imiquimod-adjuvanted influenza subunit vaccine enhances IgG2a isotype responses. Sera collected on days 14 and day 28 after immunization with adjuvanted influenza subunit vaccine were tested for virus-specific immunoglobulins by ELISA as described in materials and methods. Data are represented as arithmetic mean +/− standard deviation. Total IgG was detected by using HRP-conjugated goat anti-mouse IgG (**A**). IgG subclasses (IgG1 and IgG2a) were tested using sera collected on day 28 post-immunization. IgG subclasses were detected using HRP-conjugated goat anti-mouse IgG1 or IgG2a (**B**). (n = 9) *** - p<0.001.

To test the effect of co-delivery of poly(I:C), imiquimod, or a combination of both adjuvants with a licensed influenza subunit vaccine, female BALB/c mice (6 weeks old) were vaccinated by coated microneedles with 1 µg H1N1 HA and 1 µg of each adjuvant. On day 14, 100% (6/6) of animals seroconverted, and by day 28 IgG titers were equivalent in all vaccinated groups. The serum antibody levels indicate that microneedle delivery of influenza subunit vaccine induces antibodies against the homologous virus. Furthermore, co-delivery of imiquimod or poly(I:C) alone or in combination did not significantly enhance the serum IgG response (Figure 1A).

Adjuvants are capable of influencing the adaptive immune response and inducing a type 1 helper T cell phenotype resulting in isotype switching to IgG2a [25]. Influenza specific IgG1 and IgG2a were measured in sera collected from mice on day 28 after microneedle vaccination with H1N1 vaccine alone or with imiquimod or poly(I:C) alone or in combination. The data indicate a robust IgG1 response in all vaccinated groups. However, addition of imiquimod resulted in a significant increase in virus specific IgG2a (p = 0.0002). The IgG2a subtype is associated with the type 1 helper T cell phenotype and enhanced virus neutralization [26]. Thus, although microneedle delivery of H1N1 subunit vaccine with imiquimod did not induce a quantitatively different serum IgG response, it did induce a qualitatively different response (Figure 1B).

### Effects of Poly(I:C) and Imiquimod on HAI Titers

**Figure 2:**
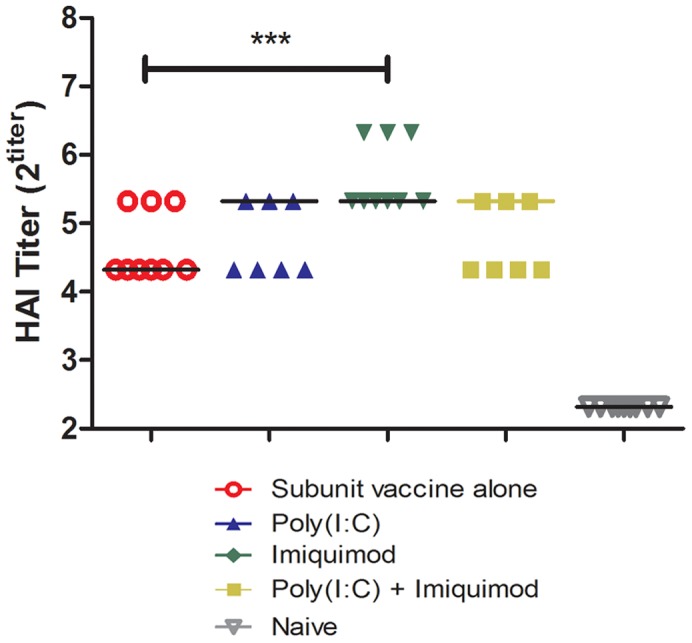
Imiquimod co-delivered with influenza subunit vaccine on coated microneedles induces higher HAI titers compared to vaccine alone. Sera collected on day 28 after immunization were tested for HAI titers as described in materials and methods. HAI titers are reported as the reciprocal of the last dilution of serum inhibiting agglutination of red blood cells. Data represented as median value. (n = 9) *** - p<0.001.

Because we detected a qualitative difference in the immunoglobulin isotypes following vaccination with and without imiquimod, we measured the titer of functional antibodies by using a hemagglutination inhibition (HAI) assay. In general, an HAI titer of ≥40 correlates with protection in humans [27]. Serum collected on day 28 from mice vaccinated with H1N1 alone or with imiquimod or poly(I:C) alone or in combination were tested for HAI titers. All mice vaccinated with H1N1 subunit vaccine and imiquimod (9/9) had HAI titers greater than or equal to 40, whereas approximately 33% of mice receiving H1N1 vaccine alone had HAI titers greater than or equal to 40. In addition, mice vaccinated with H1N1 subunit vaccine and imiquimod had approximately 2-fold higher HAI titers compared to mice vaccinated with H1N1 alone vaccine (p<0.001). Interestingly, the combination of poly(I:C) and imiquimod did not further boost the HAI titers compared to the imiquimod alone (p = .22) (Figure 2). Therefore, co-delivery of imiquimod with influenza subunit vaccine induces higher titers of functional HAI antibodies compared to vaccine alone.

### Microneedle Vaccination with Influenza Subunit Vaccine and Imiquimod Induces Higher Frequency of IFN-γ+CD4+ T Cells

**Figure 3:**
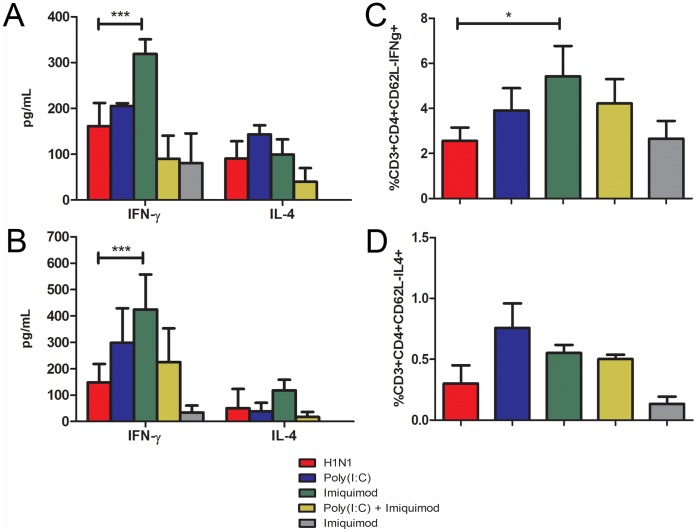
Microneedle immunization with imiquimod and influenza subunit vaccine elicits a robust IFN-γ cellular immune response. Purified T cells from the spleen and total lymph node cells were stimulated for 4 days with 5 µg of influenza subunit vaccine supplemented with 30 U/mL of recombinant human IL-2. All data represented as arithmetic mean +/− standard deviation. IFN-γ and IL-4 were detected in cell culture supernatants by ELISA (**A** - purified T cells, **B -** draining lymph node). On day 4, stimulated cells from the draining lymph node were fixed and stained for surface markers and intracellular IFN-γ (**C**) and IL-4 (**D**). (n = 3) * - p<0.05, *** - p<0.001.

T cell-derived cytokines can influence the B cell and CD8+ T cell response. [28], [29]. Therefore to determine the helper T cell phenotype, on day 14 post-vaccination we isolated the inguinal lymph nodes and purified T cells from the spleens of vaccinated mice (n = 3) and restimulated the cells with vaccine being presented by accessory cells. The cytokines IFN-γ and IL-4 are known to be produced by helper T cell type 1 and helper T cell type 2, respectively. Stimulated cells were assayed for the production of these cytokines by ELISA and intracellular cytokine staining. Mice vaccinated with subunit vaccine and imiquimod had approximately 2-fold higher IFN-γ secretion levels in stimulated splenic T cells (p = 0.01) and approximately 2.8-fold higher levels in the draining lymph node (p = 0.03) (Figure 3 A,B). In addition, we observed approximately 1.6-fold higher IL-4 secretion from stimulated splenic T cells from mice immunized with vaccine and poly(I:C) compared to mice immunized with vaccine alone (p = 0.101), while vaccination with imiquimod increased IL-4 expression approximately 2.3-fold over vaccine alone in the draining lymph node (p = 0.23). Intracellular staining of the stimulated draining lymph node cells indicated that microneedle vaccination with influenza subunit vaccine and imiquimod resulted in a 2.1-fold higher frequency of CD4+CD62L-IFNγ+ T cells (p = 0.02) and a 1.7-fold higher frequency of CD4+CD62L-IL4+ T cells compared to vaccine alone (p = 0.05)(Figure 3 C,D). These data indicate that the addition of adjuvants with the microneedle vaccine increases the frequency of type 1 helper T cells. In addition, these results support the IgG isotype data we obtained, suggesting that this increased frequency of IFN-γ+ helper T cells plays a role in inducing the IgG2a isotype after immunization with the imiquimod adjuvanted subunit vaccine.

### Immunization with Influenza Subunit Vaccine and Imiquimod-coated Microneedles Induces Improved Protective Immune Responses

**Figure 4:**
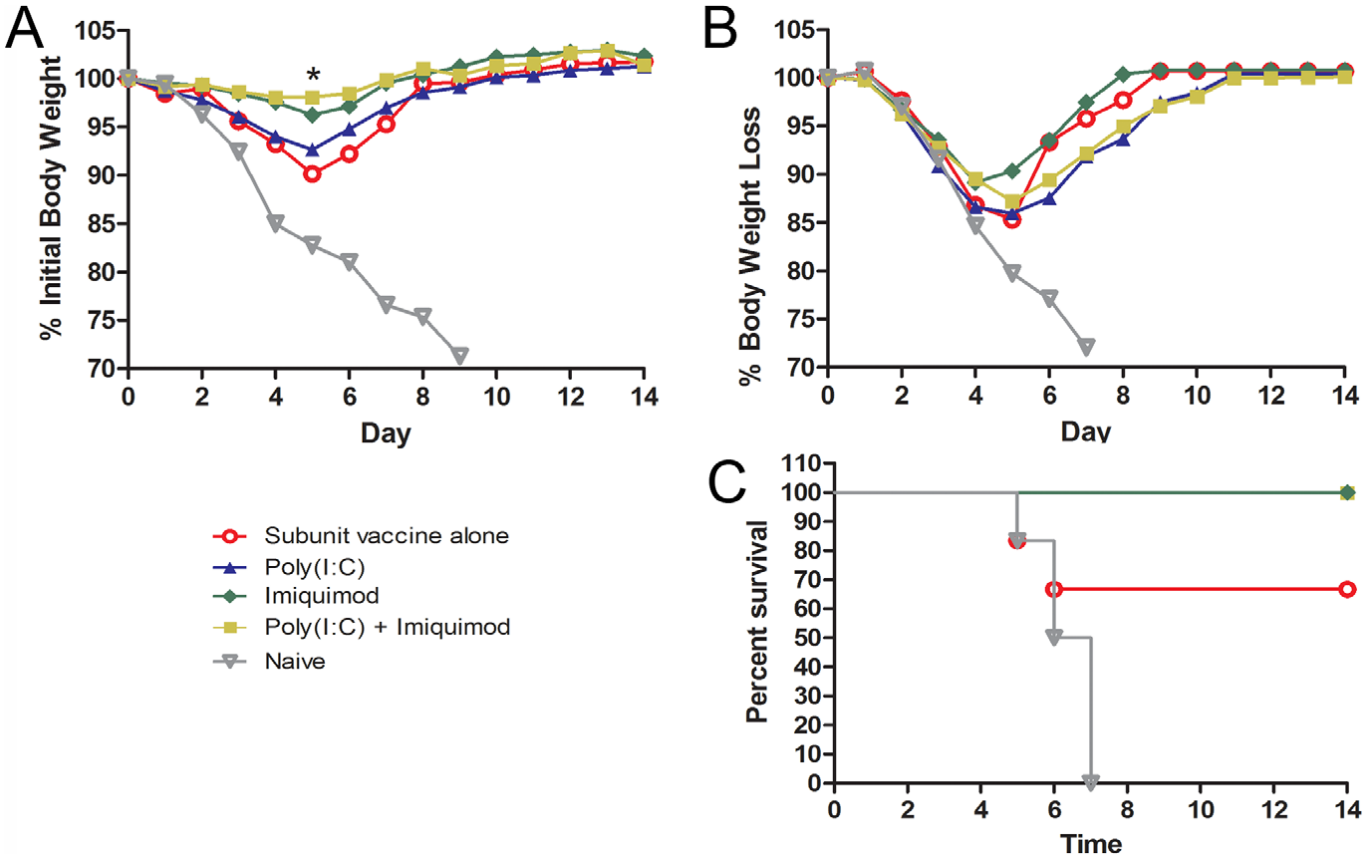
Microneedle immunization with imiquimod and influenza subunit vaccine confers enhanced protection against lethal challenge with mouse adapted A/California/07/2009 virus. Mice immunized with adjuvanted influenza subunit vaccine or vaccine alone were challenged with 5×LD_50_ (**A**) or 20×LD_50_ (**B,C**) of the mouse adapted A/California/07/2009 virus. (n = 6) * - p<0.05.

To determine the efficacy of the adjuvanted influenza subunit microneedle vaccines, we challenged vaccinated mice intranasally with 5×LD_50_ or 20×LD_50_ of the mouse adapted A/California/07/2009, 28 days after immunization. Body weights and survival were monitored for 14 days post-challenge. Following lethal challenge with 5×LD_50_, mice immunized with subunit vaccine and imiquimod or imiquimod and poly(I:C) in combination survived infection resulting in a maximal average body weight loss of approximately 2% and 4%, respectively, by day 5. Mice immunized with subunit vaccine alone or with poly(I:C) lost approximately 10% and 8%, respectively, while all naïve mice succumbed to infection. This difference in retention of body weight following lethal challenge was statistically significant when imiquimod-adjuvanted vaccine is compared to subunit vaccine alone (p = 0.04,p = 0.01)(Figure 4A).

Following lethal challenge with 20×LD_50_, mice vaccinated with subunit vaccine alone lost approximately16% of their initial body weight by day 5 and showed a survival rate of 66% (4/6). Notably, all groups of mice that were immunized with adjuvanted influenza vaccine survived the higher lethal challenge, with imiquimod providing the lowest body weight loss (10%)(Figure 4 B,C). Thus addition of imiquimod to microneedle-delivered subunit influenza vaccines improves the protective immune response to a high lethal challenge dose.

### Imiquimod Enhances Viral Lung Clearance and Reduces Production of Pro-inflammatory Cytokines

**Figure 5:**
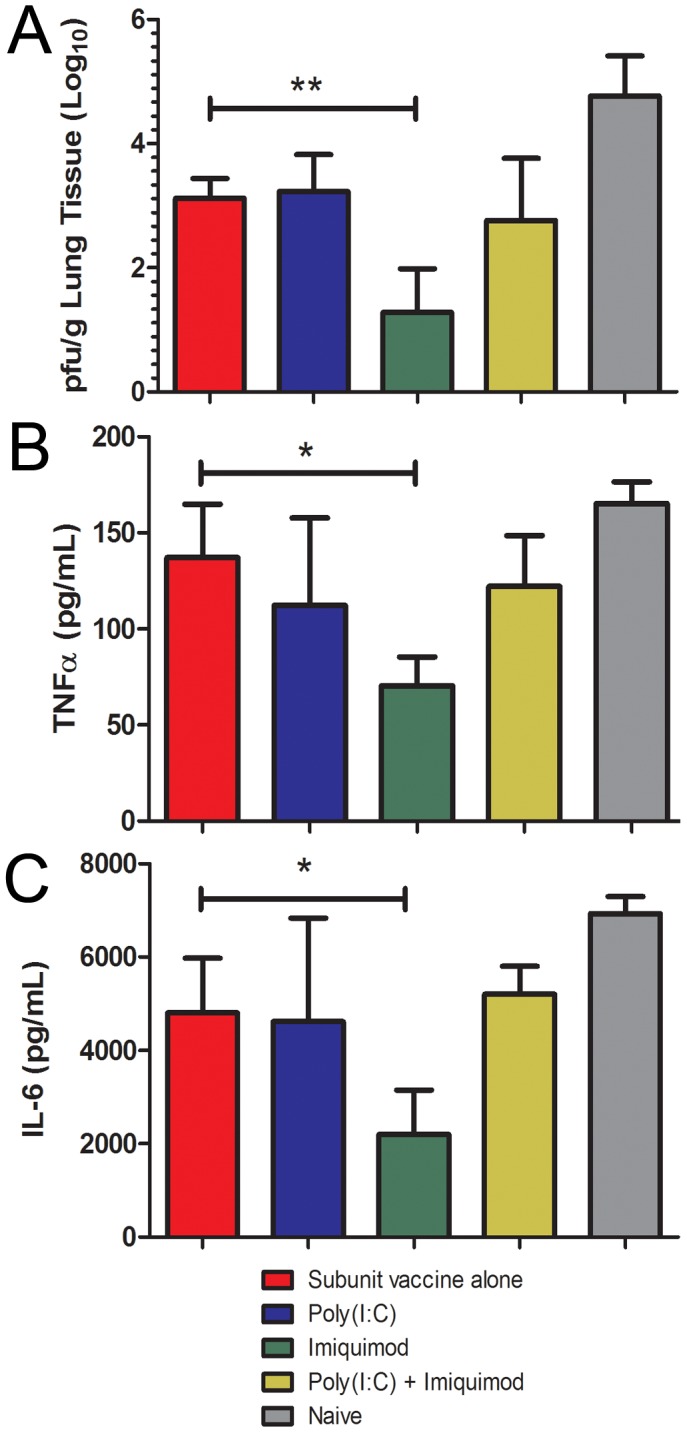
Imiquimod induces improved clearance of replicating virus from the lung and reduced production of pro-inflammatory cytokines in response to challenge. Mice immunized with adjuvanted influenza subunit vaccine or vaccine alone were challenged with 20×LD_50_ of mouse adapted A/California/07/2009 virus. All data are represented as arithmetic mean +/− standard deviation. Lungs were removed on day 4 post-challenge and processed for plaque assay (**A**) and TNFα (**B**) and IL-6 (**C**) cytokine ELISAs. (n = 3) * - p<0.05, ** - p<0.01.

To determine the ability of mice to clear viral replication in the lungs, we examined the viral lung titers 4 days post-challenge with 20×LD_50_ of the homologous virus. Mice vaccinated with the subunit vaccine and imiquimod exhibited enhanced clearance of virus with an approximately 2.5-log greater reduction in virus titers compared to subunit vaccine alone (p = 0.01). In contrast, addition of poly(I:C) to the vaccine formulation did not significantly improve clearance of virus from the lungs compared to the subunit vaccine alone (p = 0.79)(Figure 5A).

The production of pro-inflammatory cytokines in the lungs in response to influenza infection has been attributed to cellular infiltration and tissue damage. In addition, this cytokine storm is associated with morbidity in humans [30], [31]. To determine the expression of inflammatory mediators we determined the expression levels of TNFα and IL-6 in the lungs after 20×LD_50_ challenge. We observed a significant reduction in levels of both cytokines in mice immunized with subunit vaccine and imiquimod versus vaccine alone using coated microneedles (p = 0.02, p = 0.04) (Figure 5B,C). This suggests that the addition of imiquimod to the influenza subunit microneedle vaccine allows for rapid clearance of replicating virus in the lungs and reduces the level of the cytokine storm, resulting in reduced immunopathology.

## Discussion

The use of adjuvants with influenza vaccines has been shown to induce improved immune responses [32]–[35]. However, these approaches have relied on the use of traditional needle-and-syringe delivery methods. Skin-based vaccination strategies using microneedle delivery systems are an attractive alternative and take advantage of the large population of innate immune cells including dermal dendritic cells and Langerhans cells. In addition, this vaccine delivery method has other advantages including a painless vaccination [36].

In the present study, we tested the hypothesis that microneedle delivery of the TLR7 and TLR3 ligands imiquimod and poly(I:C), respectively, to the skin with a licensed monovalent influenza H1N1 subunit vaccine will enhance the immune response in mice, compared to microneedle immunization with vaccine alone. We demonstrated that although the total IgG response was not significantly enhanced following microneedle immunization using influenza vaccine and adjuvant coated microneedles compared to vaccine alone, there were significant increases in the induction of IgG2a and functional antibodies blocking receptor binding using adjuvanted subunit vaccine and microneedle delivery.

CD4+ T cells play an important role in inducing class switching in B cells. In addition to induction of class switching to IgG2a, IFN-γ has also been shown to induce an anti-viral state, and upregulate MHC class I and II [37], [38]. Therefore we measured the expression of a Th1 (IFN-γ) and a Th2 (IL-4) cytokine in mice receiving adjuvanted microneedle vaccines. We observed that vaccination with influenza subunit vaccine and imiquimod resulted in a higher frequency of IFN-γ+ effector CD4+ T cells compared to mice immunized with subunit vaccine alone. This correlated with the induction of IgG2a in the sera of mice vaccinated with subunit vaccine and imiquimod.

Further investigation into the protective immune responses indicated that adjuvanted microneedle subunit vaccine improved resistance to lethal challenge compared to vaccine only. Notably, microneedle vaccination with subunit vaccine and imiquimod resulted in improved clearance of virus from lungs. Previous studies using the 1918 H1N1 virus and highly pathogenic avian H5N1 virus suggest that a strong induction of pro-inflammatory cytokines in the lungs results in immunopathology [30], [39], [40]. We observed a reduction in pro-inflammatory cytokines in the lungs of mice that were immunized with subunit vaccine and imiquimod, which correlated with reduced body weight loss. These data suggest that microneedle vaccination with influenza subunit vaccine and imiquimod is capable of reducing immunopathology associated with infection.

It is interesting to note that the tissue expression pattern of TLRs varies greatly in mice. In murine skin, previous studies have demonstrated that isolated Langerhans cells express TLRs 2,4, and 9 mRNA [41]. Fujita et al have demonstrated that murine Langerhans cells expressed TLR3 mRNA; however they exhibited low responsiveness to poly(I:C) [42]. In our studies, delivering the TLR3 ligand, poly(I:C) using the coated microneedles did not enhance the immune response, in agreement with these findings. In addition, a recent study by Hasan et al [43] suggests that responses to topical poly(I:C) can be inhibited by anti-microbial peptides. These antimicrobial peptides are upregulated in response to disruption of the skin barrier, suggesting that the lack of a response to poly(I:C) could be due in part to this response [44].

Conversely, TLR7 has not been shown to be expressed in the skin at steady state conditions [41]. However, Suzuki et al have demonstrated that as a topically applied cream imiquimod induces migration of Langerhans cells from the skin and to the draining lymph nodes [45]. Thus, the delivery of both vaccine antigen and imiquimod by coated microneedles allows for direct uptake of vaccine by Langerhans cells and TLR7-dependent enhancement of migration to draining lymph nodes.

Recent work by our lab investigated microneedle delivery of the A/Brisbane/59/2007 H1N1 subunit vaccine and compared immune responses to intramuscular vaccination [46]. The results indicated that microneedle vaccination with 3 µg of A/Brisbane/59/2007 induced higher serum IgG2a levels compared to intramuscular vaccination at 4 weeks post-immunization. Furthermore, at 12 weeks post-immunization a higher frequency of IFN-γ secreting splenocytes was observed in mice vaccinated with coated microneedles compared to mice vaccinated intramuscularly suggesting a Th1 cytokine environment. Our present results with 1 µg of A/California/07/2009 antigen complement these results and suggest increased dose-sparing with similar body weight loss at 5×LD_50_ and improved survival after 20×LD_50_ challenge. The addition of MF59 has been shown to enhance immune responses to the California/07/2009 in adults receiving a single intramuscular vaccination with 3.75 µg of subunit vaccine [47]. In the mouse model, MF59 adjuvanted A/California/07/2009 (0.5 or 1 µg) subunit vaccine was also more immunogenic compared to vaccine alone. [48]. Although MF59 has been shown to be a very effective adjuvant, it is probably unsuitable to be coated onto solid microneedles due to its lipid content.

These results demonstrate the efficacy of vaccination using microneedles coated with influenza subunit vaccine and imiquimod. The imiquimod-adjuvanted vaccine induced greater functional antibody titers and enhanced cellular responses, inducing improved protective immune responses in mice. It is noteworthy that imiquimod is currently FDA-approved for human use when applied to the skin. In addition to TLR agonist adjuvants, other mediators of innate immunity including cytokines need to be investigated further. Taken together our results indicate that the use of adjuvants with coated-microneedle vaccines is a feasible approach for at enhancing the anti-viral immune response.

## Materials and Methods

### Influenza Vaccine and Adjuvants

H1N1 monovalent subunit influenza vaccine (A/California/07/2009) was kindly provided by Novartis Vaccine and Diagnostics (Boston, MA). Polyinosinic-polycytidylic acid (poly(I:C)) and imiquimod were purchased from Invivogen (San Diego, CA).

### Microneedle Fabrication and Coating

Microneedles were fabricated from stainless steel sheets (SS 304, 50 µm thick, Trinity Brand Industries – Countryside, IL) by lithography and wet etching. Individual microneedles had a length of 750 µm and width of 200 µm, and were assembled in rows of five microneedles each.

Microneedles were coated using a solution composed of 1% (w/v) carboxymethylcellulose sodium salt (low viscosity, USP grade, Carbo-Mer - San Diego, CA), 0.5% (w/v) Lutrol F-68 NF (BASF - Mt. Olive, NJ), soluble HA protein at 5 mg/ml and, unless otherwise noted, 15% (w/v) trehalose (Sigma – St. Lois, MO) [49]. In order to obtain a high vaccine concentration in the coating solution, we used evaporation for 5–10 minutes at room temperature (23°C) at the final step of preparation (Vacufuge - Eppendorf, NY). The coating step was performed by a dip coating process [50]. The apparatus had a chamber with coating solution and microneedle holder which was attached to a linear stage that allowed the microneedle array to move in two dimensions with 0.4 µm accuracy. The coating was automated and was monitored by a video camera attached to a computer.

To measure the amount of vaccine coated per row of microneedles, three rows out of each batch of coated microneedles were submerged into 200 µL of PBS buffer for 5 minutes to dissolve the antigen. The concentration of protein in the solution was measured by the BCA protein assay (Pierce - Rockford, IL) and was found to be consistent to ±10% within each batch.

### Vaccinations

Female BALB/c mice (6–8 weeks) were anesthetized with a xylazine/ketamine cocktail intraperitoneally and hair on the lower back removed with a depilatory cream (Nair, Church & Dwight Co. – Colonial Heights, VA) two days prior to microneedle vaccination. Mice were anesthetized again and microneedle arrays coated with 1 µg of subunit influenza vaccine alone or with 1 µg imiquimod, 1 µg poly(I:C) alone or the combination of both adjuvants were inserted into the skin and left in place for 5 minutes to allow the vaccine coating to dissolve. All animal studies were approved by the Emory University Institutional Animal Care and Use Committee (IACUC).

### Virus and Challenge

To determine vaccine efficacy, vaccinated mice were lightly anesthetized with isofluorane (4%) until recumbent and challenged by slow intranasal inoculation of 30 µL containing 5×LD_50_ or 20×LD_50_ of live mouse adapted A/California/07/2009 (H1N1) virus. Body weight loss and survival rates were monitored daily for 14 days post-challenge. Weight loss ≥25% was used as the end-point at which mice were euthanized according to IACUC guidelines.

### Viral Lung Titers and Inflammatory Cytokine ELISA

Mouse lungs were collected on day 4 post-challenge as previously described [15]. Madin-Darby canine kidney cells were maintained at a low passage number in Dulbecco’s modified Eagle’s medium supplemented with 10% fetal bovine serum (Hyclone, ThermoFisher - Rockford, IL). Plaque assays were performed on lung homogenates from challenged mice as previously described [51]. Lung supernatants in DMEM prepared from immunized mice on day 4 post-challenge were assayed for IL-6 and TNF-α according to the manufacturer’s instructions (eBioscience – San Diego, CA).

### ELISA

IgG ELISA was performed on serum and lung homogenates as previously described [15], [17]. All horseradish-peroxidase (HRP) conjugated secondary antibodies to mouse IgG, IgG1, and IgG2a were purchased from Southern Biotechnology Associates (Birmingham, AL). Briefly, 96-well immunoplates (Nunc, Co - Rochester, NY) were coated overnight at 4°C with 4 µg of inactivated A/California/07/2009 virus per well. Plates were washed with PBS-Tween (0.05%) and blocked with PBS-Tween supplemented with 3% BSA. Sera were diluted 1∶100 and incubated for 1.5 hours at 37°C. Plates were washed 3 times with PBS-Tween (0.05%) and incubated for 1.5 hours at 37°C in a 1∶1000 dilution of goat, anti-mouse HRP-conjugated secondary antibody (Southern Biotechnology – Birmingham, AL). Plates were then washed 3 times with PBS-Tween (0.05%) and OPD substrate (Invitrogen – Carlsbad, CA), added to each well and color allowed to develop for 10 minutes. Color development was stopped using 1 M phosphoric acid. Absorbances were read at 450 nm on a Biorad Model 680 microplate reader. IgG concentrations were determined by linear regression of a standard curve of known concentrations. Absorbances for IgG1 and IgG2a were corrected for background (no antigen).

### Hemagglutination Inhibition Assay (HAI)

HAI tests were performed on vaccinated animal sera based on the World Health Organization protocol [52]. Briefly, sera were treated with receptor destroying enzyme (Denka Seiken, Tokyo, Japan) for 16 hours at 37°C then heat inactivated for 30 minutes at 56°C. Treated sera were diluted to a final concentration of 1∶10 in PBS and incubated with packed chicken red blood cells (RBC) for 1 hour at 4°C to remove cryoglobulins. Treated sera were serially diluted and incubated with 4 HA units of A/California/07/2009 virus for 30 minutes at room temperature. An equal volume of 0.5% chicken RBC was added to each well and incubated for 30 minutes at room temperature. The HAI titer was read as the reciprocal of the highest dilution of serum that inhibited hemagglutination. Values were expressed as the geometric mean with a 95% confidence interval.

### Cellular Immune Responses

Single cell suspensions were prepared from spleens and inguinal lymph nodes of mice 14 days post vaccination or naïve mice, by mincing the tissue through a 70 µm cell strainer (BD Falcon - San Jose, CA). Splenocytes preparations were incubated in red blood cell lysing buffer (Sigma) to remove RBCs. T cells were purified by negative selection using BD iMag magnetic cell separation (BD Biosciences – San Jose, CA). Naïve splenocytes were treated with Mitomycin C for 30 minutes and used as accessory cells (2.5×10^5^ cells/well) after incubation with 5 µg of vaccine in complete RPMI overnight at 37°C in a 5% CO_2_ incubator in a 96-well round bottom microtiter plate (Nunc, Co – Rochester, NY). Purified T cells or lymph node cells were added to accessory cells at a ratio of 4∶1 and 3∶1 (responder:accessory), respectively, in complete RPMI medium supplemented with 30 U of recombinant human IL-2 (BD Biosciences). In vitro antigen-specific stimulation was measured after incubation for 5 days by intracellular cytokine staining. Supernatants of stimulated cells were collected and clarified by centrifugation for IFN-γ and IL-4 ELISA, performed according to the manufacturer’s instructions (eBioscience – San Diego, CA).

### Antibodies and Flow Cytometry

Cells were washed with PBS, 1% BSA buffer and surface stained with fluorochrome-conjugated antibodies to CD4, CD62L, and CD3, followed by intracellular staining of IFN-γ and IL-4. Antibodies were purchased from eBiosciences (San Diego, CA) and BD Biosciences. For intracellular cytokine staining, cells were fixed and permeabilized using the BD Cytofix/Cytoperm manufacturer’s protocol and reagents (BD Bioscience). The data were acquired on a BD LSR-II and analyzed with FloJo Software (v7.6.1, Tree Star – Ashland, OR).

### Statistics

Statistical analysis was done using the one-way ANOVA analysis with GraphPad Prism5 software (La Jolla, CA). All data followed normal Gaussian distributions unless otherwise noted. Viral lung titers and HAI titers were transformed to log_10_ and log_2_ titers, respectively, for statistical analysis using one-way ANOVA. A P value less than 0.05 was considered as a significant difference.
